# Research progress on the application of fascial plane blocks in postoperative pain management in spinal surgery

**DOI:** 10.3389/fmed.2025.1714286

**Published:** 2025-12-09

**Authors:** Huixia Wang, Yan Liu, Li Luo, Cheng Tang, Zhengqi Chang, Huan Liu

**Affiliations:** 1 Department of Anesthesiology, The 960th Hospital of PLA, Jinan, Shandong, China; 2 Department of Anesthesiology, The 961th Hospital of PLA, Qiqihar, Heilongjiang, China; 3 Department of Orthopedics, The 960th Hospital of PLA, Jinan, Shandong, China

**Keywords:** thoracolumbar fascia plane block, spinal surgery, postoperative pain relief, ultrasound guidance, block

## Abstract

Postoperative pain after spine surgery (POPSS) is a critical issue that affects patient recovery and quality of life, increasing the risk of complications such as thrombosis. The side effects of traditional pain management methods, such as intravenous opioid administration, limit their clinical application. Therefore, it has become essential to explore and investigate new pain management strategies. We conducted a review of the relevant literature on fascial plane blocks (FPBs) in postoperative pain management after spine surgery, sourced from CNKI and PubMed, with a search period from June 25, 2020, to April 20, 2025. Additionally, we manually examined the references of the included studies to identify any potentially overlooked literature. The review findings indicate that FPBs are a novel regional anesthesia technique and a key component of multimodal analgesia. With precise analgesic effects, ease of operation, high safety, and few complications, FPBs have become a research focus in postoperative pain management for spine surgery. This article provides a comprehensive review of the characteristics of postoperative spine pain, the anatomical basis of FPBs, clinical research progress, controversial studies and limitations, as well as future research directions, aiming to offer valuable references for both clinical practice and research.

## 1 Background

More than 50% of patients undergoing spinal surgery experience moderate to severe acute pain, which significantly affects their quality of life and postoperative recovery (1). Traditional opioid analgesics are commonly associated with side effects, including respiratory depression, drug dependence, and nausea and vomiting (2). Fascia plane blocks (FPBs) are effective in blocking pain signal transmission, reducing the need for opioid medications, improving patient safety and comfort, and supporting the overarching goal of enhanced recovery after surgery (ERAS).

In recent years, the widespread adoption of ultrasound-guided technology has further facilitated the application of FPBs in spinal surgery, optimizing their effectiveness. This article aims to evaluate the efficacy and safety of FPBs in postoperative pain management following spinal surgery, offering enhanced analgesic strategies for clinical practice.

## 2 Characteristics of postoperative spinal pain and analgesic requirements

### 2.1 Pain mechanism

Spinal fusion surgery, discectomy, and spinal decompression are common spinal procedures that often lead to significant postoperative pain. This pain can have a profound impact on patients’ physical and mental well-being, as well as hinder their recovery. If not properly managed, it may progress to chronic pain, further complicating the recovery process.

The mechanisms underlying postoperative pain following spinal surgery are multifactorial and complex. Current understanding suggests several contributing factors: somatic pain resulting from surgical trauma, inflammatory pain caused by paraspinal muscle dissection, and neuropathic pain due to nerve root traction (3). The primary mechanisms of pain involve sensitization in both the peripheral and central nervous systems. Surgical trauma induces tissue injury, which activates nociceptors and leads to peripheral sensitization. Additionally, the release of pro-inflammatory cytokines such as interleukin-6 (IL-6) and interferon-alpha (INF-α) from damaged tissues and immune cells plays a key role. These inflammatory factors activate glial cells in the dorsal spinal cord, contributing to central sensitization and resulting in heightened pain sensitivity (hyperalgesia). This process not only amplifies the intensity of pain but may also contribute to the persistence of pain beyond the acute postoperative period.

A thorough understanding of these pain mechanisms is essential for developing effective analgesic strategies that target both peripheral and central pain pathways, thereby improving postoperative outcomes and reducing the risk of chronic pain development.

### 2.2 Limitations of traditional pain relief methods

Spinal surgery often leads to severe postoperative pain and significant opioid consumption. Studies indicate that approximately 57% of patients experience inadequate pain control following surgery (4). The most commonly used analgesic approach for spinal surgery remains the intravenous administration of opioid medications. However, 30–40% of patients experience side effects such as respiratory depression, nausea, and vomiting (2), which significantly detract from their quality of life. Moreover, opioid abuse has become a public health crisis, further complicating perioperative pain management.

The accelerated ERAS protocol emphasizes multimodal analgesia (MMA) as a strategy to reduce opioid use, shorten hospital stays, decrease postoperative complications, and accelerate recovery. This approach not only focuses on improving surgical outcomes but also addresses pain management, promotes early functional recovery, and enhances patient satisfaction (5).

Neuraxial nerve blocks are gaining attention for their ability to provide rapid and sustained analgesia, reducing the need for opioids in the postoperative period. They offer significant potential advantages in the management of pain following spinal surgery (1). However, despite their benefits, neuraxial blocks are technically complex, expensive, and can lead to postoperative complications, including urinary retention, skin itching, epidural hematoma, infection, and motor blockage. Further research is needed to validate the long-term efficacy and safety of this technique in clinical practice.

These challenges highlight the need for continued innovation in pain management strategies, aiming to minimize opioid reliance while optimizing patient comfort and recovery.

## 3 Clinical application of FPBs in the treatment of spinal surgical pain

In recent years, with the widespread adoption of multimodal perioperative analgesia strategies and ultrasound-guided techniques, nerve blocks have become increasingly common in postoperative pain management, expanding their scope of application significantly. FPBs have shown considerable advantages in perioperative pain management due to their high safety profile, ease of administration, and reliable analgesic effects (6, 7). These benefits have drawn significant attention from researchers in recent years, particularly in clinical applications (7–11). FPBs have been widely utilized in areas such as bariatric surgery, joint replacement surgery, and laparoscopic procedures (12–14). Furthermore, in the field of spinal surgery, FPBs are beginning to demonstrate their unique advantages (15).

### 3.1 Anatomical basis of FPBs

Fascia is a complex collagenous connective tissue with a unique microscopic structure, containing loose connective tissue that facilitates the diffusion of local anesthetics. These drugs can reach pain receptors and neurons through bulk flow and diffusion, reducing muscle stiffness, alleviating pain, and improving the range of motion (16). Fascia is also rich in blood vessels and lymphatics (17, 18), which contribute to both local and systemic analgesic effects, thereby enhancing the overall analgesic efficacy (19). FPBs achieve analgesia by injecting local anesthetics into specific fascia planes, blocking nerves within those planes, or allowing the anesthetic to diffuse into paravertebral spaces, where it can block spinal nerve roots and branches (6, 20). Additionally, systemic analgesia can occur as local anesthetics are absorbed into the bloodstream (20–24), further improving overall pain relief for patients (25). The direct relationship between the microscopic structure of fascia and its analgesic mechanism, with both its microscopic and macroscopic anatomical features contributing to effective analgesia, makes understanding this mechanism essential for optimizing pain management (26). However, the specific mechanisms of action of FPBs are not yet fully understood and continue to be the subject of ongoing research.

### 3.2 Clinical application of thoracolumbar interfascial plane block (TLIP)

TLIP block was first introduced by Hand in 2015 and later modified by Ahiskalioglu et al. (27). It is a novel interfascial plane block technique designed to block the dorsal branches of thoracolumbar spinal nerves (28), and it is widely used for perioperative analgesia in lumbar spine surgery. The traditional method involves inserting the needle from the outside inwards, injecting local anesthetic into the fascial space between the longissimus muscle and the multifidus muscle. The modified technique, however, involves inserting the needle from the inside outwards, injecting the drug into the space between the longissimus muscle and the iliocostalis muscle. Numerous clinical studies have shown (29) that both methods effectively block the dorsal branches of the spinal nerves and their branches, providing reliable analgesia during surgery. Ciftci et al. (30) found that both approaches provided significant analgesic effects, with no differences in visual analogue scale (VAS) scores, intraoperative and postoperative opioid consumption, or the need for rescue analgesia. However, the modified technique had a shorter procedure time and a higher first-attempt success rate. Ahiskalioglu et al. (27) suggested that compared to traditional approaches, the modified TLIP block route reduces the risk of extensive epidural anesthesia. With the puncture site positioned farther from the surgical incision, the risk of infection is also lowered. Additionally, ultrasound imaging facilitates the identification of the fascia between the longissimus muscle and the iliocostalis muscle, which enhances the success rate of the modified TLIP block (27). Therefore, while the modified method offers comparable analgesic efficacy to the traditional method, the iliocostalis muscle is more easily identified than the multifidus muscle, and the inward-to-outward puncture technique enhances safety.

Hu et al. (31) confirmed the efficacy of TLIP for postoperative analgesia after lumbar surgery in a meta-analysis of nine randomized controlled trials (RCTs). Within 24 h postoperatively, the TLIP group showed significantly lower VAS scores for both movement and rest, reduced PCA button-pressing frequency, decreased PCA consumption, and a lower incidence of nausea compared to the control group. Results from a single-center RCT showed that TLIP significantly reduced postoperative pain following lumbar interbody fusion surgery. At 24 hours postoperatively, TLIP decreased resting VAS scores by 1.6 points and dynamic VAS scores by 1.1 points, while also significantly reducing anesthetic consumption (32). The results of an RCT conducted by Eltaher et al. (33) suggested that TLIP reduces intraoperative pain severity, mitigates hemodynamic fluctuations, and lowers the incidence of postoperative complications. TLIP used in transforaminal lumbar interbody fusion (TLIF) surgery provides adequate intraoperative and postoperative pain relief, promoting rapid recovery (34, 35). The study by YANG Xiaolin et al. demonstrated that ultrasound-guided TLIP combined with extracoporeal shock wave is more effective than either TLIP or extracoporeal shock wave alone in treating chronic non-specific low back pain. These findings offer new insights and approaches for managing chronic NLBP in pain clinics and support the broader adoption of this combined treatment in clinical practice (36).

**Figure 1:**
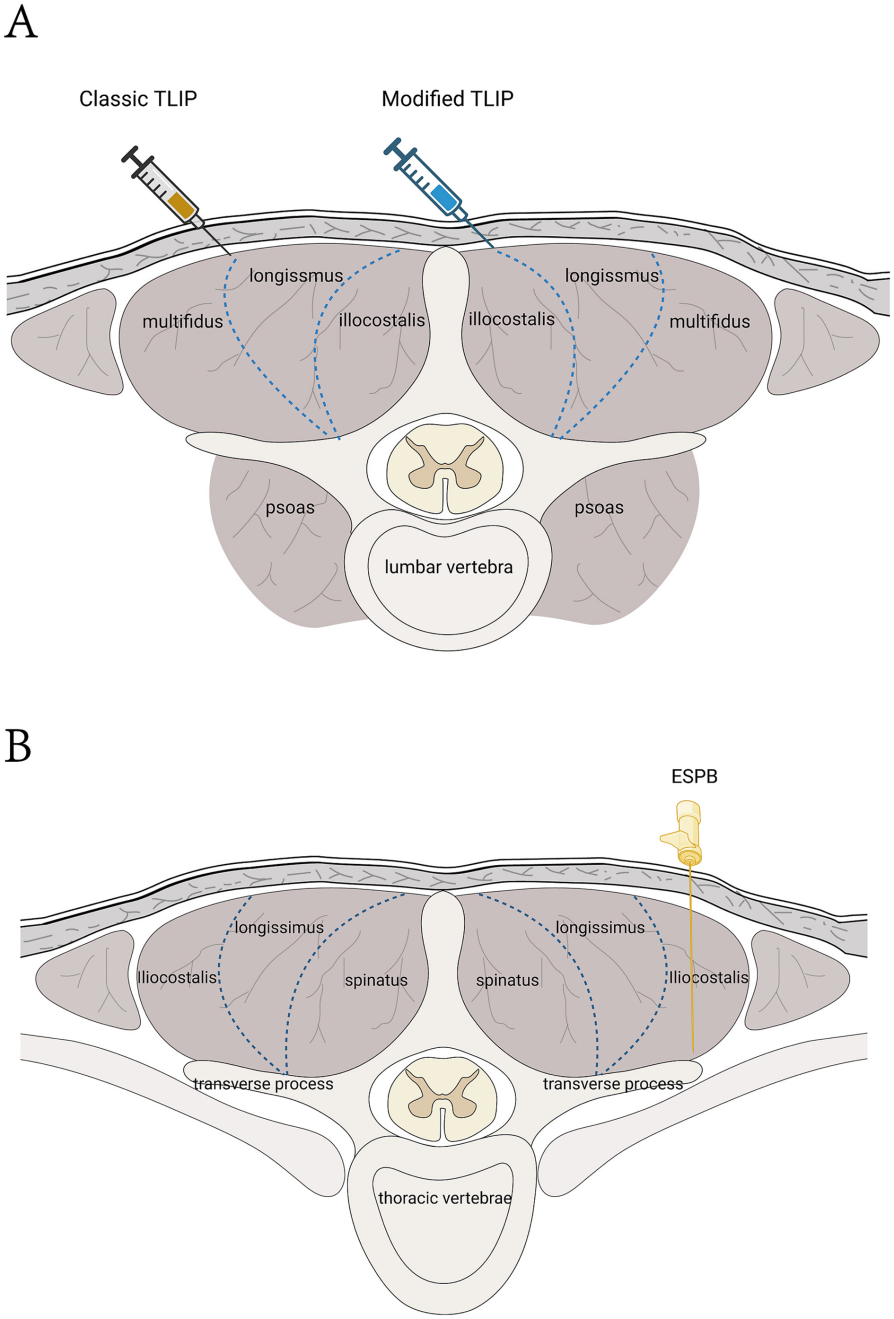
**(A)** Schematic diagram of the TLIP. In the classic TLIP, the target gap lies between the multifidus and longissimus muscles, with the direction extending from the exterior to the interior. In the modified TLIP, the target gap is positioned between the longissimus and iliocostalis muscles, with the direction extending from the interior to the exterior. **(B)** ESPB schematic diagram. The needle insertion target is the deep surface of the erector spinae muscle, located between the transverse processes.

**Figure 2:**
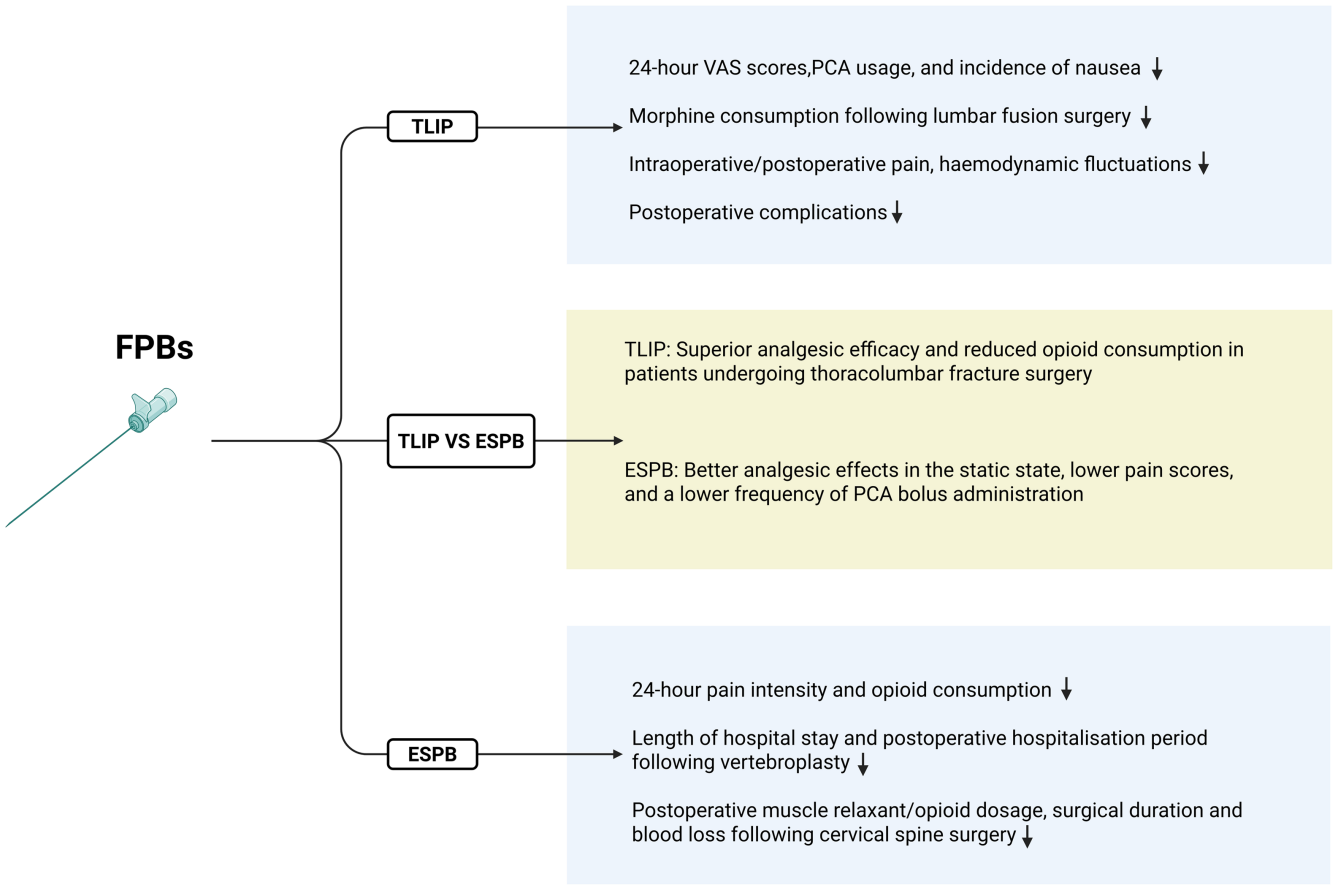
This figure demonstrates the respective advantages of TLIP and ESPB.

Numerous retrospective studies, case reports, and RCTs have confirmed the efficacy of TLIP in postoperative pain management in spinal surgery. Ultrasound-guided techniques can precisely avoid damage to nerves, blood vessels, and vital organs. TLIP is easy to perform and is an important component of multimodal pain management for postoperative spinal surgery patients. However, these studies have some limitations, including small sample sizes, inconsistent research methodologies, variations in the types, concentrations, and dosages of local anesthetics, and limited reporting of complications. The efficacy and safety of TLIP require further confirmation through large-scale, multi-center, double-blind controlled trials (see Figures 1A, 2).

### 3.3 Erector spinae plane block (ESPB)

ESPB is a novel FPB technique that involves injecting local anesthetic drugs between the deep surface of the erector spinae muscle and the transverse processes. This technique blocks both the ventral and dorsal branches of the spinal nerves to achieve analgesic effects (37). The absorption of local anesthetics into the bloodstream can produce a general anesthetic effect, while the immunomodulatory properties of the anesthetics also contribute to ESPB’s analgesic effects (38, 39). Additionally, the diffusion of local anesthetics into the paravertebral tissues further extends their analgesic reach. Additionally, the diffusion of local anesthetics into the paravertebral tissues further extends their analgesic reach. ESPB is performed away from vital organs and blood vessels, reducing the risk of complications such as pneumothorax and hematoma. As a result, it is widely used for perioperative analgesia across various surgical fields (40), effectively alleviating postoperative acute pain and providing excellent analgesic outcomes. It has gained particular attention in the management of postoperative pain in spinal surgery (41, 42).

Liu et al. (43) confirmed the efficacy and safety of ESPB for patients undergoing lumbar spine surgery through a meta-analysis of RCTs. This analysis included 19 RCTs involving 1,561 patients, and the results demonstrated that the ESPB group significantly reduced postoperative pain intensity and opioid consumption within 24 h after surgery. A randomized double-blind controlled study examined the efficacy of ESPB for postoperative pain management following posterior cervical spine surgery. The study found that ESPB reduced the use of muscle relaxants during surgery, decreased opioid consumption during and within 24 h postoperatively, shortened surgical duration, reduced blood loss, and resulted in significantly lower Numerical Rating Scale (NRS) scores at all time points within 72 h postoperatively compared to the control group (44). Additionally, ESPB has proven to be an effective anesthetic technique for vertebroplasty in high-risk patients, and it has been associated with a shorter length of stay in the intensive care unit and a reduced postoperative hospital stay (45). ESPB is now a commonly used neuroblockade technique for perioperative pain management in spinal surgery. However, most of the existing studies are case reports and retrospective analyses with small sample sizes and limited reports of complications. There is a need for further multi-center, large-sample, double-blind controlled trials to validate its clinical efficacy and safety profile (see Figures 1B, 2).

### 3.4 Comparison between ESPB and TLIP

A meta-analysis by Liu et al. (43) demonstrated that ESPB can reduce opioid consumption during the perioperative period, alleviate both resting and movement-related postoperative pain, shorten hospital stays, decrease the incidence of nausea and vomiting, and improve patient satisfaction. However, it did not show any clear advantage over the TLIP block. Ciftci et al. (30) conducted a randomized controlled trial comparing ESPB and TLIP in 90 patients undergoing lumbar discectomy. Both methods provided effective postoperative analgesia for lumbar surgery, and there were no statistically significant differences in analgesic efficacy or procedure time between the two techniques. In a larger randomized controlled trial involving 304 patients undergoing lumbar surgery, Wang et al. (46) found that compared to TLIP, ESPB offered better analgesia in static conditions, with lower pain scores and reduced PCA bolus frequency. This could be attributed to the broader coverage of ESPB. On the other hand, Kim et al. (47) compared postoperative analgesic efficacy and PCA usage between the TLIP and ESPB groups. The results showed that the modified TLIP group had lower VAS scores, fewer effective PCA presses, and reduced sufentanil consumption compared to the ESPB group. These findings suggest that the modified TLIP block provides superior analgesia compared to ultrasound-guided ESPB in thoracolumbar spine fracture surgery. This advantage may be attributed to the fact that, although ESPB covers a wider area, the thickness and volume of the erector spinae muscles can, to some extent, limit the effectiveness of local anesthetics in the surgical region.

Overall, while both techniques offer benefits, further high-quality RCTs are needed to directly compare the two methods and elucidate their respective advantages (see Figure 2).

## 4 Controversies and limitations of FPB clinical studies

Although the block technique is relatively simple to perform, it requires the physician to have significant expertise in ultrasound-guided needle placement. Additionally, its application may be limited in certain patient populations, such as those with coagulation disorders or spinal deformities (48). This could be due to factors like unclear thoracolumbar fascia layers and anatomical variations in the fascia. Research suggests that practitioners must be especially cautious of anatomical variations in thoracic spine deformities, as drug diffusion within non-standard fascial layers may differ from that in conventional anatomical structures. This variation could impact the efficacy of drug diffusion (49). FPBs exhibit instability in head-to-tail diffusion, and low-volume ESPBs can result in a more extensive sensory plane block. The range of forward diffusion varies significantly and is not universally agreed upon. Additionally, drug diffusion shows considerable individual variability, meaning that ESPBs of the same volume at the same level can produce different sensory block planes in different patients. The success rate of ESPB and the depth of sensory block are influenced by factors such as operator technique, the targeted transverse process, the concentration and volume of local anesthetic, and patient-specific characteristics (50). For patients undergoing spinal correction surgery that requires neural function monitoring, FPBs may mask signals from motor evoked potentials and somatosensory evoked potentials, requiring caution during such procedures.

There is currently no unified consensus or guideline on the optimal puncture site, drug dosage, and volume, all of which can influence the efficacy of FPBs and the occurrence of complications. The choice of injection site often depends on the operator’s experience. Common local anesthetics used in clinical practice include ropivacaine and bupivacaine, typically at concentrations of 0.25, 0.375, and 0.5%, with volumes ranging from 10 to 30 mL. Identifying the optimal drug concentration and volume that balances effectiveness with the risk of complications, such as local anesthetic toxicity, remains a key area of research.

Long-term data on rare complications such as local anesthetic toxicity and nerve damage are insufficient, with follow-up periods rarely exceeding 1 year. As such, further clinical validation is necessary to better understand the risks and benefits of FPBs. The high cost of ultrasound equipment and training also limits the widespread use of FPBs in primary care hospitals, and the collaborative approach between anesthesiology and orthopaedics has yet to be fully implemented.

Different patient inclusion criteria often lead to selection bias, and factors such as varying pain sensitivity and metabolic differences among elderly patients can impact the efficacy of FPBs. Additionally, there is limited research on FPBs in pediatric populations, warranting further investigation. Outcome measures such as pain scores contain subjective elements, which may influence the results of studies. Current research primarily includes prospective cohort studies, retrospective analyses, RCTs, and case reports, which can lead to varying conclusions. Furthermore, small sample sizes, short follow-up periods, and a lack of blinded protocols may also affect the validity of results.

In conclusion, multi-center, large-scale RCTs are essential to clarify the applicability of FPBs in different surgical contexts and to establish their long-term safety, cost-effectiveness, and overall clinical utility.

## 5 Research directions and outlook

With the growing focus on comfort-oriented medical care, novel FPB techniques have emerged as key areas of research in perioperative pain management. Multimodal pain management protocols, which combine FPBs with various analgesic drugs of different mechanisms, are expected to become a standard approach in spinal surgery, facilitating safe and rapid postoperative recovery. The ERAS protocol with regional anesthesia via TLIP block significantly reduces length of stay and opioid requirements in patients undergoing lumbar spinal surgery (51). The advent of ultrasound visualization technology has accelerated the development of FPBs. In the future, artificial intelligence-assisted ultrasound image recognition may enable real-time monitoring of local anesthetic diffusion, reducing puncture error rates and improving precision. Liposomal bupivacaine, composed of bupivacaine and a lipophilic carrier, allows for a sustained and controlled release of the drug into surrounding tissues. When used in lumbar fusion surgery, liposomal bupivacaine is safe and effective in reducing postoperative pain for up to 72 hours, minimizing perioperative opioid use, and promoting earlier postoperative recovery (52). Additionally, technologies like spinal cord stimulators and neurophysiological monitoring are becoming integrated into perioperative pain management, promising further improvements in patient comfort and postoperative recovery.

## 6 Summary

As spinal surgery techniques advance and our understanding of pain mechanisms deepens, pain management is increasingly tailored to individual patients. FPBs offer a precise method for blocking pain pathways, significantly reducing the reliance on opioid medications after spinal surgery. Their effectiveness and safety have been validated by numerous clinical trials, making them a promising option for postoperative pain management. FPBs have become a core technique in MMA, presenting a new paradigm for managing pain after spinal surgery.

However, challenges remain, including the need for technical standardization, safety validation, and the development of individualized treatment protocols. Further research is essential to address these issues, clarify the clinical efficacy of FPBs, and minimize potential complications. Ultimately, these efforts will help meet the growing demand for patient-centered care in pain management.
