# Supplementary material for: Research progress on the application of fascial plane blocks in postoperative pain management in spinal surgery
Source: Front Med (Lausanne). 2025 Dec 9;12:1714286. doi: 10.3389/fmed.2025.1714286 (PMC12722993; doi:10.3389/fmed.2025.1714286)

Pubmed Search Query：18

| Search number | Query | Results |
| --- | --- | --- |
| 1 | Spinal surgery[Title/Abstract] | 9,700 |
| 2 | Lumbar spine surgery[Title/Abstract] | 2,062 |
| 3 | Thoracic spine surgery[Title/Abstract] | 122 |
| 4 | ((Spinal surgery[Title/Abstract]) OR (Lumbar spine surgery[Title/Abstract])) OR (Thoracic spine surgery[Title/Abstract]) | 11,578 |
| 5 | Plane Blocks[Title/Abstract] | 826 |
| 6 | (((Spinal surgery[Title/Abstract]) OR (Lumbar spine surgery[Title/Abstract])) OR (Thoracic spine surgery[Title/Abstract])) AND (Plane Blocks[Title/Abstract]) | 18 |

CNKI Search Query：208

Chinese search term：（主题：筋膜平面阻滞）OR（主题：竖脊肌平面阻滞）AND（主题：腰椎）OR（主题：胸椎）OR（主题：脊柱）AND（主题：腰椎）OR（主题：胸椎）OR（主题：脊柱）

English search terms：(Subject: Fascial Plane Block) OR (Subject: Erector Spinae Plane Block) AND (Subject: Lumbar Spine) OR (Subject: Thoracic Spine) OR (Subject: Spine) AND (Subject: Lumbar Spine) OR (Subject: Thoracic Spine) OR (Subject: Spine)


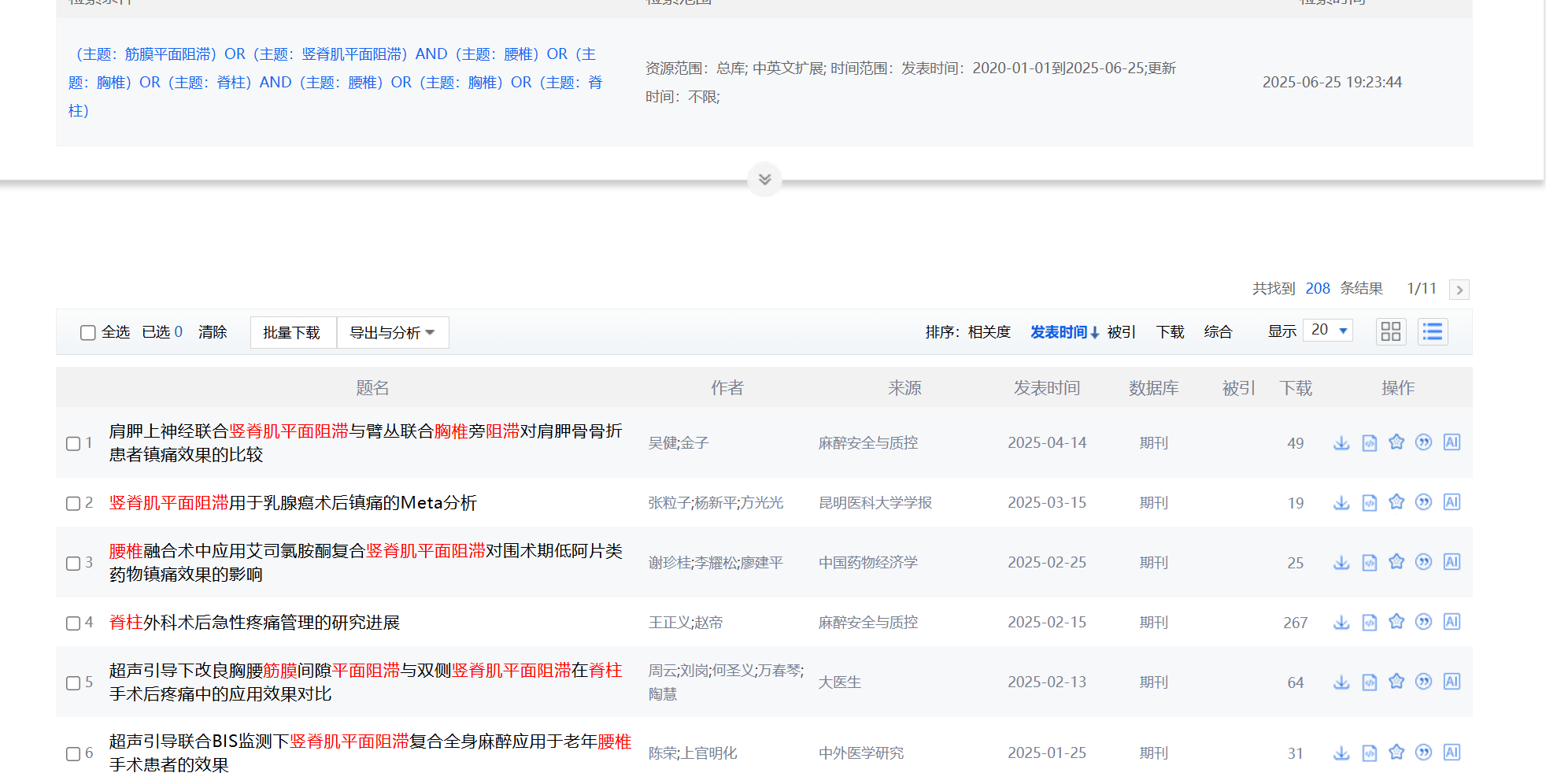

Supplement: Supplementary file 1 [file Table_1.docx]
